# Topological Optimization of Phononic Crystal Thin Plate by a Genetic Algorithm

**DOI:** 10.1038/s41598-019-44850-8

**Published:** 2019-06-06

**Authors:** X. K. Han, Z. Zhang

**Affiliations:** State Key Laboratory of Structural Analysis for Industrial Equipment, Department of Engineering Mechanics, Faculty of Vehicle Engineering and Mechanics, Dalian University of Technology, Dalian, 116024 China

**Keywords:** Mechanical engineering, Topological insulators

## Abstract

Genetic algorithm (GA) is used for the topological optimization of phononic crystal thin plate composed of aluminum and epoxy resin. Plane wave expansion (PWE) method is used for calculations of band gaps. Fourier displacement property is used to calculate the structure function in PWE. The crossover rate and the mutation rate are calculated according to the adaptive GA method. Results indicate that filling rates, symmetry, polymerization degree and material parameters are key factors for design of topological configurations. The relations between the key factors and different topologies are studied in detail.

## Introduction

As a kind of periodic composite materials, phononic crystal (PC) has been noticed widely and studied by more and more scholars due to its rich physical properties in controlling the propagation of elastic waves^1–8^. The elastic wave band gaps (BGs) is a significant feature of PCs. Because of the influence of the PC periodicity, the special frequency range between two bands that have no intersection is called the band gap, in which the elastic wave cannot propagate through the PC. The band gaps produced by PC is valuable both in theory and in practice for sound insulation^9–12^. According to the formation of BGs, PCs can be divided into two categories, Bragg scattering or the local resonance^13–15^. For the Bragg scattering PC, the wavelength relevant to the band gap and lattice constant are of the same order of magnitude. For the local resonance PC, the wavelength relevant to the band gap is much greater than the lattice constant.

The changes of lattice types and material parameters can affect the BGs of PC^16–20^. How to obtain the new configurations with wider BGs is the key factor for design of PC. Topology optimization is a method to show how to place material within a prescribed design domain in order to obtain the best structural performance^21^ and can be used to design material distributions in PC. Initial contribution to topological optimization of PC comes from Sigmund *et al*.^22^. The finite element method with the method of moving asymptotes is adopted to optimize the PC. The multiple genetic algorithm method with the adaptive fuzzy fitness granulation^23^ can be also used for topological optimization in PC. The procedure converges much faster than the conventional algorithm. The bi-directional evolutionary structural optimization algorithm^24^ are more efficient due to the sensitivity analysis of the double eigenvalues. For one-dimensional PC unit cell^25^, optimization is mainly focused on the design of material thickness. But for two-dimensional case^26^, material distribution becomes the main factor which needs to be designed for optimization. Topological optimization can be further applied to metal matrix composite systems and fluid-filled porous media^27^. The optimization method mentioned above can also be used to design the PC for unidirectional acoustic transmission^28^ by maximizing the minimum imaginary part of the wave vector in a specific direction instead of creating the widest BG directly.

The topological methods like the homogenization method^29^ and evolutionary structure method^30^ are widely used for many useful designs. Compared with the traditional optimization method, genetic algorithm is selected due to its self-organization and self-study strategies for optimization^31,32^. When genetic algorithm runs by use of the information obtained in the evolutionary process, the individuals with higher fitness have higher survival probability and more adaptive gene structures can be obtained.

The plane wave method is one of the most widely used algorithms in the calculation of the band structure or field distribution because of its fast convenience^33,34^. Elastic constants, density and other parameters can be expanded by Fourier series because the PCs are periodic. Combined with Bloch’s theorem, the elastic wave equation can be expanded as superposition of plane waves in the reciprocal space. The band structure of PC is obtained by solving the eigen-equation transformed from the wave equation.

The manufacturing issues can affect the final properties of PC. At present, manufacturing methods of metamaterials mainly includes Fused Deposition Modeling (FDM)^35,36^, Stereo Lithography Apparatus (SLA)^37,38^, Selected Laser Sintering/Melting (SLS/SLM)^39,40^, Direct Laser Writing (DLW)^41,42^ and Polymer Injection Technology (PIT)^43,44^. The manufacturing issues can affect the final properties. However, theoretical test verification and functional device applications are fundamental issues for PC design regardless of manufacturing types. So, the current research is still focusing on theoretical test verification and functional device applications. Significant achievements have been made by combination of topological optimization with PC design. However, the topologies of PCs can be affected by the given parameters for design, e.g. the filling rates, symmetry, polymerization degree and material parameters. So, it is necessary to investigate how the given material parameters affect the final topology of PC. In this paper, the optimization design of the PC thin plate is studied when the influence factors are considered to obtain the maximum relative band gap based on the plane wave expansion method and the genetic algorithm. The unit cell is divided into N × N elements and each element is filled with epoxy resin or aluminum randomly. The optimal topological configuration of the PC plate can be obtained. The key factors, including filling rates, symmetry, polymerization degree and material parameter for the generation of band gaps, are correlated with different optimal topologies. By this design method on key factors including the filling rates, symmetry, polymerization degree and material, the optimal PC with much wider band gap features on given conditions can be obtained.

## Model and Method

### Analysis of the thin PC plate

**Figure 1:**
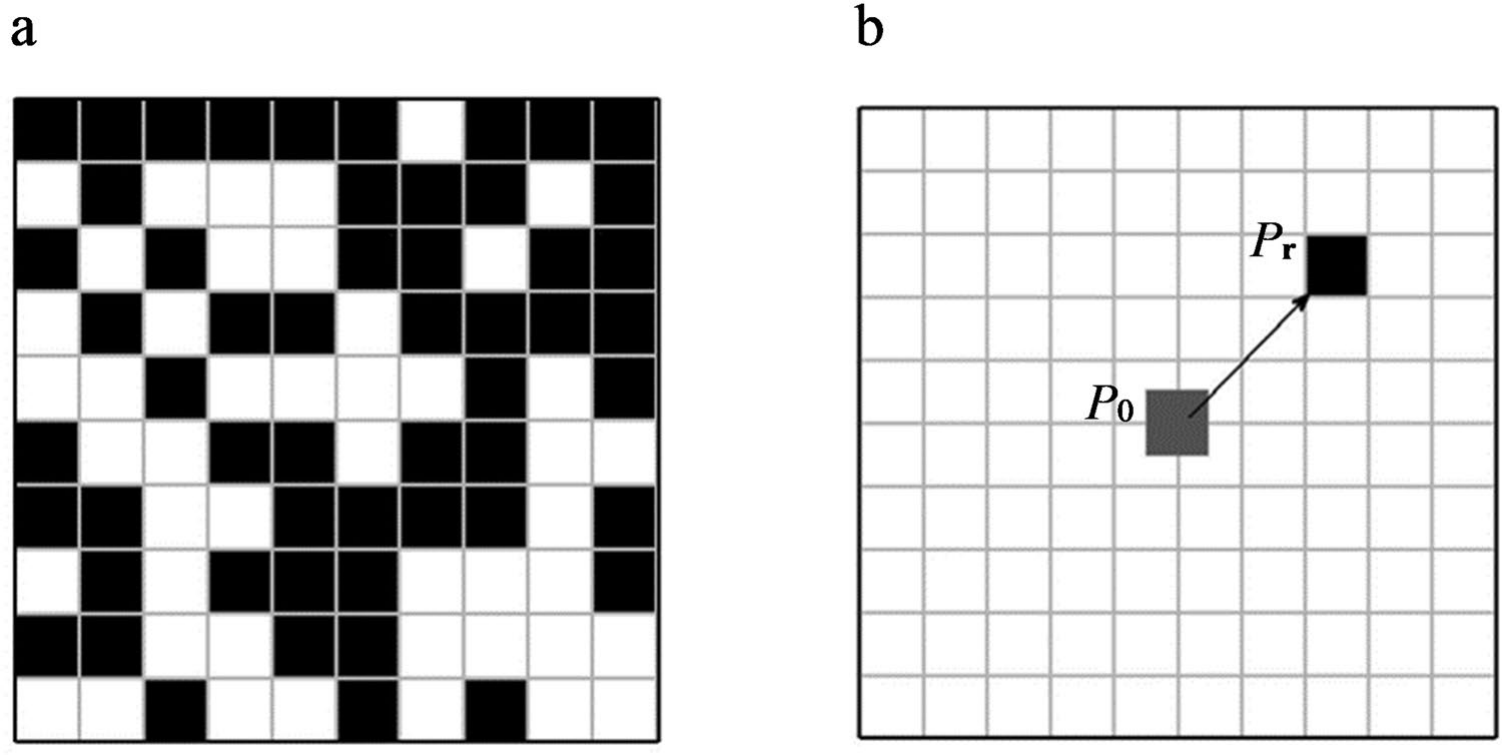
The unit cell. (**a**) 10 × 10 cell structure (**b**) displacement characteristic.

A 2D PC thin plate is discussed. The lattice constant is *a* = 0.02 m. As we can see in Fig. 1(a), the unit cell is a square lattice which is divided into 10 × 10 elements. Epoxy resin and aluminum are chosen as the material of the substrate and the scatterer respectively. The material parameters of the epoxy and aluminum are given as follows: the density *ρ*_e_ = 1180 kg/m^3^, the elasticity modulus *E*_e_ = 0.435 × 10^10^ Pa and the Poisson’s ratio *v*_e_ = 0.159 for epoxy resin; the density *ρ*_a_ = 2730 kg/m^3^, the elasticity modulus *E*_a_ = 7.76 × 10^10^ Pa and the Possion’s ratio *v*_a_ = 2.87 for aluminum.

For each element in unit cell, the material parameters are defined as:

1a $$\rho ({x}_{i})=(1-{x}_{i}){\rho }_{{\rm{a}}}+{x}_{i}{\rho }_{{\rm{e}}}$$

1b $$E({x}_{i})=(1-{x}_{i}){E}_{{\rm{a}}}+{x}_{i}{E}_{{\rm{e}}}$$

1c $$\nu ({x}_{i})=(1-{x}_{i}){\nu }_{{\rm{a}}}+{x}_{i}{\nu }_{{\rm{e}}}$$

where *i* ∈[1, 2, 3, …100], *x*_*i*_ = 0 or 1.

The study of the thin plate can be regarded as the plane stress state because the thickness of the plate is much less than the lattice constant. When the elastic wave propagates in the thin plate, the stress-strain relation can be expressed as^45^:

2a $${\sigma }_{xz}={\sigma }_{yz}={\sigma }_{zz}=0$$

2b $${\sigma }_{xx}=\frac{E}{1-{\nu }^{2}}(\frac{\partial {u}_{x}}{\partial x}+\nu \frac{\partial {u}_{y}}{\partial y})$$

2c $${\sigma }_{yy}=\frac{E}{1-{\nu }^{2}}(\frac{\partial {u}_{y}}{\partial y}+\nu \frac{\partial {u}_{x}}{\partial x})$$

2d $${\sigma }_{xy}=\frac{E}{2(1+\nu )}(\frac{\partial {u}_{x}}{\partial y}+\frac{\partial {u}_{y}}{\partial x})$$

The longitudinal wave equation is^45^:

3a $$\rho \frac{{\partial }^{2}{u}_{x}}{\partial {t}^{2}}=\frac{\partial {\sigma }_{xx}}{\partial x}+\frac{\partial {\sigma }_{xy}}{\partial y}$$

3b $$\rho \frac{{\partial }^{2}{u}_{y}}{\partial {t}^{2}}=\frac{\partial {\sigma }_{yx}}{\partial x}+\frac{\partial {\sigma }_{yy}}{\partial y}$$

Bring the stress-strain relation into the wave equation:

4a $$\rho \frac{{\partial }^{2}{u}_{x}}{\partial {t}^{2}}=\alpha \frac{{\partial }^{2}{u}_{x}}{\partial {x}^{2}}+\beta \frac{{\partial }^{2}{u}_{y}}{\partial x\partial y}+\mu (\frac{{\partial }^{2}{u}_{x}}{\partial {y}^{2}}+\frac{{\partial }^{2}{u}_{y}}{\partial x\partial y})$$

4b $$\rho \frac{{\partial }^{2}{u}_{y}}{\partial {t}^{2}}=\alpha \frac{{\partial }^{2}{u}_{y}}{\partial {y}^{2}}+\beta \frac{{\partial }^{2}{u}_{x}}{\partial x\partial y}+\mu (\frac{{\partial }^{2}{u}_{x}}{\partial x\partial y}+\frac{{\partial }^{2}{u}_{y}}{\partial {x}^{2}})$$

where $$\alpha =E/(1-{\nu }^{2})$$, $$\beta =E\nu /(1-{\nu }^{2})$$, $$\mu =E/(2(1-\nu )).$$

By the plane wave expansion, the characteristic equations are obtained:

5a $$\begin{array}{l}{\omega }^{2}\sum _{{\boldsymbol{G}}^{\prime} }\rho ({\boldsymbol{G}}^{\prime\prime} -{\boldsymbol{G}}^{\prime} ){u}_{{\bf{k}}+{\bf{G}}{\boldsymbol{^{\prime} }}}^{x}\\ \begin{array}{rcl} & = & \sum _{{\boldsymbol{G}}^{\prime} }[\alpha ({\boldsymbol{G}}^{\prime\prime} -{\boldsymbol{G}}^{\prime} ){({\boldsymbol{k}}+{\boldsymbol{G}}^{\prime} )}_{x}{({\boldsymbol{k}}+{\boldsymbol{G}}^{\prime\prime} )}_{x}\\  &  & +\,\mu ({\boldsymbol{G}}^{\prime\prime} -{\boldsymbol{G}}^{\prime} ){({\boldsymbol{k}}+{\boldsymbol{G}}^{\prime} )}_{y}{({\boldsymbol{k}}+{\boldsymbol{G}}^{\prime\prime} )}_{y}]{u}_{{\boldsymbol{k}}+{\boldsymbol{G}}{\boldsymbol{^{\prime} }}}^{x}\\  & = & \sum _{{\boldsymbol{G}}^{\prime} }[\beta ({\boldsymbol{G}}^{\prime\prime} -{\boldsymbol{G}}^{\prime} ){({\boldsymbol{k}}+{\boldsymbol{G}}^{\prime} )}_{y}{({\boldsymbol{k}}+{\boldsymbol{G}}^{\prime\prime} )}_{x}\\  &  & +\,\mu ({\boldsymbol{G}}^{\prime\prime} -{\boldsymbol{G}}^{\prime} ){({\boldsymbol{k}}+{\boldsymbol{G}}^{\prime} )}_{x}{({\boldsymbol{k}}+{\boldsymbol{G}}^{\prime\prime} )}_{y}]{u}_{{\bf{k}}+{\bf{G}}{\boldsymbol{^{\prime} }}}^{y}\end{array}\end{array}$$

5b $$\begin{array}{l}{\omega }^{2}\sum _{{\bf{G}}^{\prime} }\rho ({\bf{G}}^{\prime\prime} -{\bf{G}}^{\prime} ){u}_{{\bf{k}}+{\bf{G}}{\boldsymbol{^{\prime} }}}^{y}\\ \begin{array}{rcl} & = & \sum _{{\bf{G}}^{\prime} }[\beta ({\bf{G}}^{\prime\prime} -{\bf{G}}^{\prime} ){({\bf{k}}+{\bf{G}}^{\prime} )}_{x}{({\bf{k}}+{\bf{G}}^{\prime\prime} )}_{y}\\  &  & +\,\mu ({\bf{G}}^{\prime\prime} -{\bf{G}}^{\prime} ){({\bf{k}}+{\bf{G}}^{\prime} )}_{y}{({\bf{k}}+{\bf{G}}^{\prime\prime} )}_{x}]{u}_{{\bf{k}}+{\bf{G}}^{\prime} }^{x}\\  & = & \sum _{{\bf{G}}^{\prime} }[\alpha ({\bf{G}}^{\prime\prime} -{\bf{G}}^{\prime} ){({\bf{k}}+{\bf{G}}^{\prime} )}_{y}{({\bf{k}}+{\bf{G}}^{\prime\prime} )}_{y}\\  &  & +\,\mu ({\bf{G}}^{\prime\prime} -{\bf{G}}^{\prime} ){({\bf{k}}+{\bf{G}}^{\prime} )}_{x}{({\bf{k}}+{\bf{G}}^{\prime\prime} )}_{x}]{u}_{{\bf{k}}+{\bf{G}}^{\prime} }^{y}\end{array}\end{array}$$

For the center element *P*_0_ in Fig. 1(b), the Fourier expansion coefficient of the parameters is 0 when it is filled with the epoxy resin. When *P*_0_ is filled with the aluminum, the Fourier expansion coefficient of the parameters can be expressed as:

6 $${g}_{0}({\boldsymbol{G}})=\{\begin{array}{ll}\frac{1}{100}({g}_{{\rm{a}}}-{g}_{{\rm{e}}})\frac{\sin ({G}_{x}a/2N)}{{G}_{x}a/2N}\frac{\sin ({G}_{y}a/2N)}{{G}_{y}a/2N}, & {\boldsymbol{G}}\ne 0\\ \frac{1}{100}({g}_{{\rm{a}}}-{g}_{{\rm{e}}})+{g}_{{\rm{e}}}, & {\boldsymbol{G}}=0\end{array}$$

where *g* represents the parameters *ρ*, *α*, *β*, *μ*

According to the Fourier displacement property, the Fourier expansion coefficient of the parameters of the element *P*_r_ (*P*_r_ ∈ *P*_a_, *P*_a_: the set of the elements including the aluminum) in Fig. 1(b):

7 $${g}_{{\boldsymbol{r}}}({\boldsymbol{G}})={g}_{0}(G){e}^{iGr}$$

For the unit cell:

8 $$\begin{array}{rcl}g({\boldsymbol{G}}) & = & \sum _{{\boldsymbol{r}}\in {P}_{{\rm{a}}}}{g}_{{\boldsymbol{r}}}({\boldsymbol{G}})=\sum _{{\boldsymbol{r}}}{{g}}_{{\boldsymbol{r}}}({\boldsymbol{G}})\delta ({\boldsymbol{r}})\\  & = & {g}_{0}({\boldsymbol{G}})\sum _{{\boldsymbol{r}}}{e}^{i{\boldsymbol{Gr}}}\delta ({\boldsymbol{r}})\\  & = & {g}_{0}({\boldsymbol{G}})e({\boldsymbol{G}})\cdot \delta \end{array}$$

where $${\boldsymbol{e}}({\boldsymbol{G}})=[{e}^{i{\boldsymbol{G}}\cdot {{\boldsymbol{r}}}_{1}},{e}^{i{\boldsymbol{G}}\cdot {{\boldsymbol{r}}}_{2}},\mathrm{...},{e}^{i{\boldsymbol{G}}\cdot {{\boldsymbol{r}}}_{100}}]$$, $${\boldsymbol{\delta }}=[\delta ({{\boldsymbol{r}}}_{1}),\delta ({{\boldsymbol{r}}}_{2}),\mathrm{...},\delta ({{\boldsymbol{r}}}_{100})]$$, $$\delta ({\boldsymbol{r}})=\{\begin{array}{c}1,\,{\boldsymbol{r}}\in {P}_{{\rm{a}}}\\ 0,\,{\boldsymbol{r}}\notin {P}_{{\rm{a}}}\end{array}$$.

At the same time, the method combined with PWE and GA can be extended to multi-material PC structure. The computational costs can be increased according to^46^ for optimization in multi-material case. When this method is extended to three-material, the stress-strain relation and wave equation remain unchanged. However, the material expression Eq. (1) need to be the linear combination of the material parameters and x_i_ in Eq. (1) should changes to be 0 or 1 or 2. At the same time, Eq. (8), the Fourier expansion coefficient of the parameters which can influence the calculation of the characteristic equation Eq. (5), should change to be the combination of two other materials:

9 $$\begin{array}{rcl}g({\boldsymbol{G}}) & = & \sum _{{\boldsymbol{r}}\in {P}_{1}}{g}_{{\boldsymbol{r}}}^{1}({\boldsymbol{G}})+\sum _{{\boldsymbol{r}}\in {P}_{2}}{g}_{{\boldsymbol{r}}}^{2}({\boldsymbol{G}})\\  & = & \sum _{{\boldsymbol{r}}}{g}_{{\boldsymbol{r}}}^{1}({\boldsymbol{G}}){\delta }_{1}({\boldsymbol{G}})+\sum _{{\boldsymbol{r}}}{g}_{{\boldsymbol{r}}}^{2}({\boldsymbol{G}}){\delta }_{2}({\boldsymbol{G}})\\  & = & {g}_{o}^{1}({\boldsymbol{G}})\sum _{{\bf{r}}}{e}^{i{\boldsymbol{G}}{\bf{r}}}{\delta }_{1}({\boldsymbol{G}})+{g}_{o}^{2}({\boldsymbol{G}})\sum _{{\boldsymbol{r}}}{e}^{i{\boldsymbol{Gr}}}{\delta }_{2}({\boldsymbol{G}})\\  & = & {g}_{o}^{1}({\boldsymbol{G}}){\boldsymbol{e}}({\boldsymbol{G}})\cdot {{\boldsymbol{\delta }}}_{1}+{g}_{o}^{2}{\boldsymbol{e}}({\boldsymbol{G}})\cdot {{\boldsymbol{\delta }}}_{2}\end{array}$$

where $${\boldsymbol{e}}({\boldsymbol{G}})=[{e}^{i{\boldsymbol{G}}{{\boldsymbol{r}}}_{1}},{e}^{i{\boldsymbol{G}}{{\boldsymbol{r}}}_{2}},\mathrm{...},{e}^{i{\boldsymbol{G}}{{\boldsymbol{r}}}_{L}}]$$, $${\boldsymbol{\delta }}={{\boldsymbol{\delta }}}_{1}+{{\boldsymbol{\delta }}}_{2}=[\delta ({{\boldsymbol{r}}}_{1}),\delta ({{\boldsymbol{r}}}_{2}),\ldots ,\delta ({{\boldsymbol{r}}}_{100})]$$, $$\delta ({\boldsymbol{r}})=\{\begin{array}{ll}0 & {\rm{else}}\\ 1, & {\boldsymbol{r}}\in {P}_{1}\\ 2, & {\boldsymbol{r}}\in {P}_{2}\end{array}$$.

The algorithm can also be extended to 3D PC optimization. When this method is extended to 3D PC, the governing equation Eq. (4) should be changed to add the vibration along z-direction according to^47^. So, the calculation of the band gap includes two conditions: in-plane and out-plane models. The calculation of the Fourier expansion coefficient of the parameters, Eq. (6) is also changed according to^48^ in the plane wave expansion method. For optimization, the computational costs are obviously increased when PC changes from 2D to 3D structure according to^49^.

### The genetic algorithm

The genetic algorithm starts with initializing a population of *N*p = 20 chromosomes randomly. The chromosomes are then evaluated by the plane wave expansion method. The objective function is the relative band gap (RBG) width and the optimization can be simplified as:

To find *ρ*(*x*_*i*_)

10 $${\rm{Max}}(2\frac{{{\rm{\min }}}_{k}{w}_{j+1}(k,{\bf{X}})-{{\rm{\max }}}_{k}{w}_{j}(k,{\bf{X}})}{{{\rm{\min }}}_{k}{w}_{j+1}(k,{\bf{X}})+{{\rm{\max }}}_{k}{w}_{j}(k,{\bf{X}})})$$

st. *ρ*(*x*_*i*_) = 0 or 1;

$${\rm{sum}}\,{\boldsymbol{X}}={f}\cdot 100,\,0\le f\le 1;$$

where ***X*** is the chromosome of the topological configuration, *k* is the wave vector, *j* is the number of band, *f* is the filling rate of the unit cell.

GA operation includes selection, crossover and mutation after population initiation. The roulette wheel selection algorithm, called proportional selection algorithm, is mainly used in the optimization algorithm^50^. The probability of being selected for each individual is proportional to its fitness. In this paper, we use this method to select individuals with larger fitness as the parents for next generation in the selection operation. The algorithm of the roulette is as follows:

1. Calculate the fitness *f*_*i*_ (*i* = 1, 2 …, *M*) for each individual *x*_*i*_, *M* is the population size.
2. Calculate the corresponding individual selection probability:
  $${P}_{i}=\frac{{f}_{i}}{{\sum }_{j=1\,}^{M}{f}_{j}}$$
3. Calculate individual cumulative probability:
  $${q}_{i}=\sum _{j=1}^{i}{P}_{j}$$
4. Generate a uniformly distributed random pseudorandom number *t*, *t* ∈ [0, 1].
5. If *t* ≤ *q*_1_, then the individual *x*_1_ is chosen; else, the individual *x*_*k*_ is chosen if *q*_*k*−*1*_ *<* *t* ≤ *q*_*k*_ *(2* ≤ *k* ≤ *M)*
6. Repeat the steps (4) and (5) M times.

The multi-point crossover is adopted according to the characteristics of the thin PC plate chromosome to make the population keep high diversity and *avoid trapping the* local optimum. Generation with new individuals can be produced by altering the values of some genes of the chromosome in the mutation operation. The mutation and the crossover parameter can directly affect the convergence of the algorithm. If the crossover rate Pc and the mutation rate Pm are fixed values, there are a lot of disadvantages like the destruction of the diversity of the population and the individuals with high fitness and the stagnant process. So the adaptive genetic algorithm proposed by Srinvivas^51^ is adopted. The crossover rate P_c_ and the mutation rate P_m_ can change with the fitness. The P_c_ and P_m_ increases when the population traps in local optimum. For the individual with large fitness, the P_c_ and P_m_ becomes small to make the individual move to the next generation. So the current mutation scheme from Srinvivas ensures the convergence of genetic algorithm while maintaining the diversity of population. The P_c_ and the P_m_ are defined as^51^:

11 $${P}_{{\rm{c}}}=\{\begin{array}{ll}{P}_{{\rm{c1}}}-\frac{({P}_{{\rm{c1}}}-{P}_{{\rm{c2}}})(f^{\prime} -{f}_{{\rm{avg}}})}{{f}_{{\rm{\max }}}-{f}_{{\rm{avg}}}}, & f^{\prime} \ge {f}_{{\rm{avg}}}\\ {P}_{{\rm{c1}}}, & f^{\prime}  < {f}_{{\rm{avg}}}\end{array}$$

12 $${P}_{{\rm{m}}}=\{\begin{array}{ll}{P}_{{\rm{m1}}}-\frac{({P}_{{\rm{m1}}}-{P}_{{\rm{m2}}})({f}_{{\rm{\max }}}-f)}{{f}_{{\rm{\max }}}-{f}_{{\rm{avg}}}}, & f\ge {f}_{{\rm{avg}}}\\ {P}_{{\rm{m1}}}, & f < {f}_{{\rm{avg}}}\end{array}$$

where *f*_max_ is the maximum fitness of the population, *f*_avg_ is the average fitness of the population, *f* ′ is the bigger fitness of the two individuals in crossover operation, *f* is the fitness of the individual in mutation operation, *P*_c1_ = 0.9, *P*_c2_ = 0.6, *P*_m1_ = 0.1, *P*_m2_ = 0.001.

**Figure 2:**
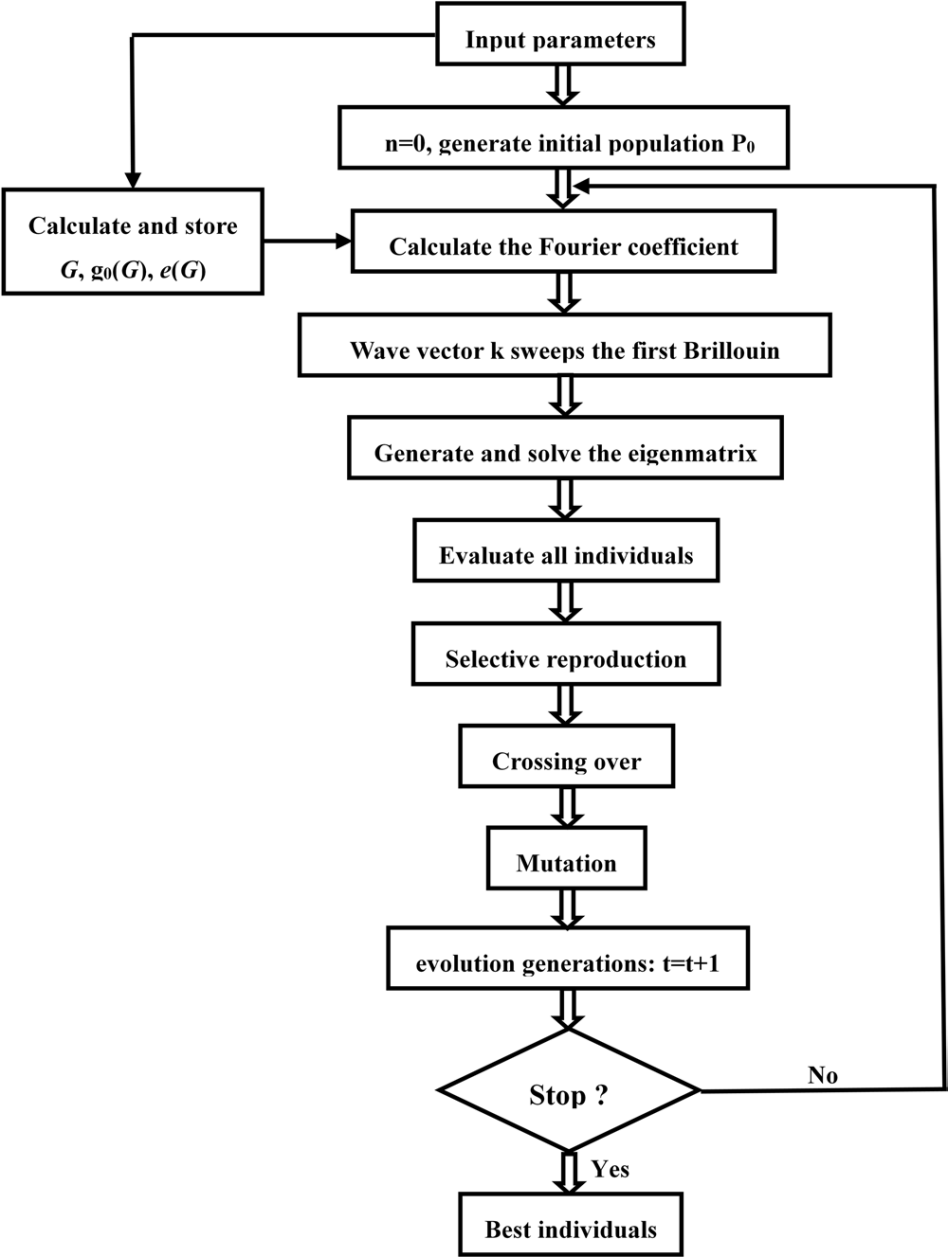
The genetic algorithm evolution flow chart.

The genetic algorithm evolution flow chart is shown in Fig. 2.

## Results and Discussions

### The influence of the filling rate

**Figure 3:**
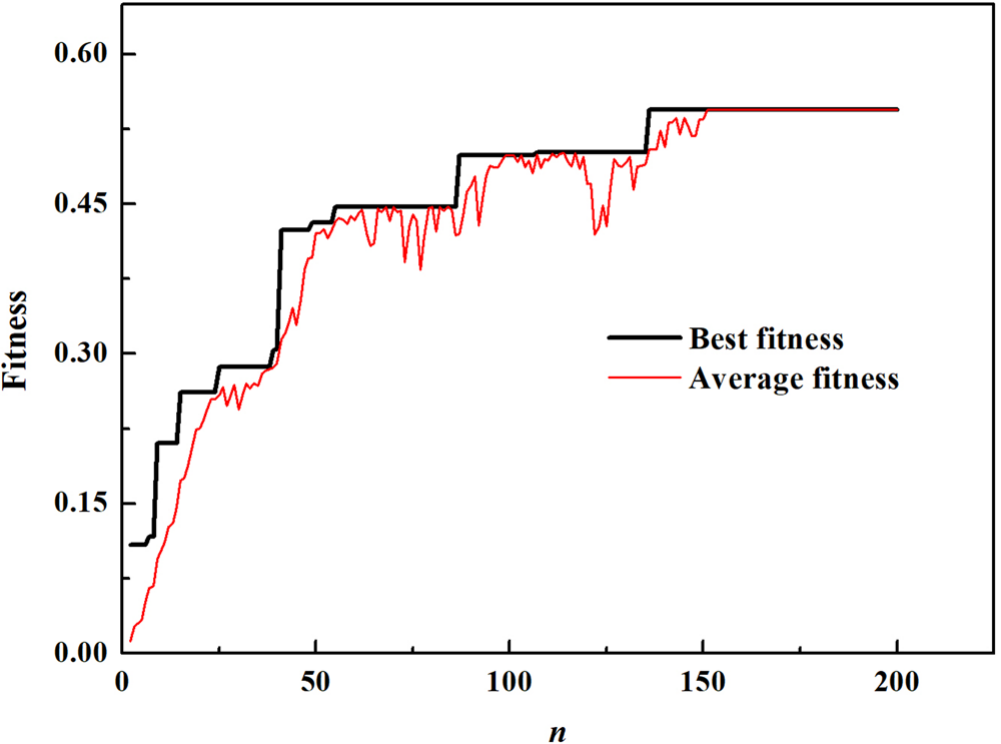
Convergence curves of the best fitness and the average fitness of evolution generation for the RBG optimization of thin PC plate.

The optimization of the relative band gap is performed when the filling rate is not fixed. The convergence criterion is the continuous equality of the best fitness and the average fitness. In fact, we have tested to calculate 500 generations. But after 150 generations, the best fitness converges a stable value of 0.56. So only 200 generations are given in Fig. 3. For each time for our calculation, the generated different random state leads to the same optimal configurations. The convergence speed is rapid in the earlier stage of the evolution and it becomes slower in the following stage. The average fitness of the first generation is 0.02 so that the BG is very small or even did not exist for the most initial individuals produced randomly in the earlier stage of the evolution. But the best fitness of the first generation is 0.1 so that the band gap of the thin PC plate opens and can be found easily.

**Figure 4:**
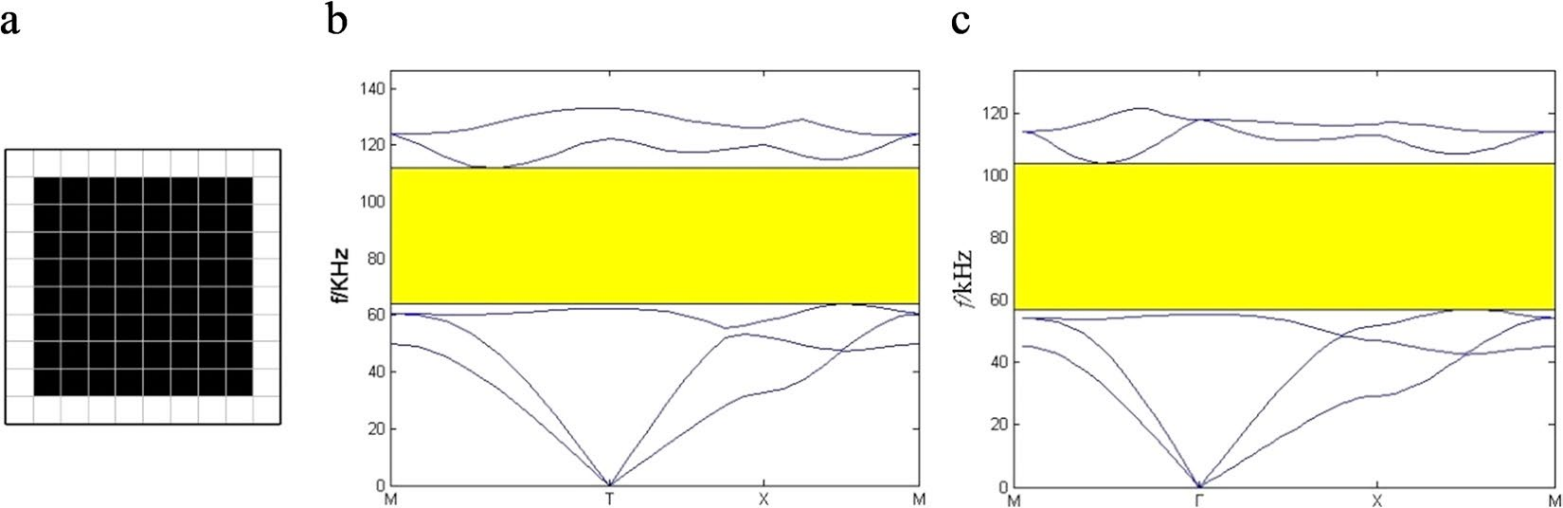
The topological optimization results. (**a**) The optimal configuration. (**b**) The corresponding band structure by GA (**c**) band structure by Comsol.

The unit cell obtained from topological optimization is shown in Fig. 4(a). It can be seen that the unit cell is a simple lattice structure with square scatterer. The corresponding filling rate of the scatterer is 0.64. The corresponding band structure is plotted in Fig. 4(b). The absolute BG is ranged from 63.9 kHz to 113.9 kHz between the third and fourth bands. The optimal RBG is 0.56. To *verify* the current algorithm, the band structures of PC thin plate is recalculated by the finite element software Comsol when the filling rate is 0.64 in Fig. 4(c). Because of the periodicity of the structure, only one unit cell is calculated. Stress-free boundary conditions are imposed on the free surfaces and the periodic boundary conditions on the two opposite boundaries of the unit cell according to the Bloch theorem. By sweeping the wave vectors in the first irreducible Brillouin zone, the Bloch calculation gives the eigen-frequencies. Then the *band structure is* obtained. The RBG is 0.58. The error is smaller than 0.05%.

**Figure 5:**
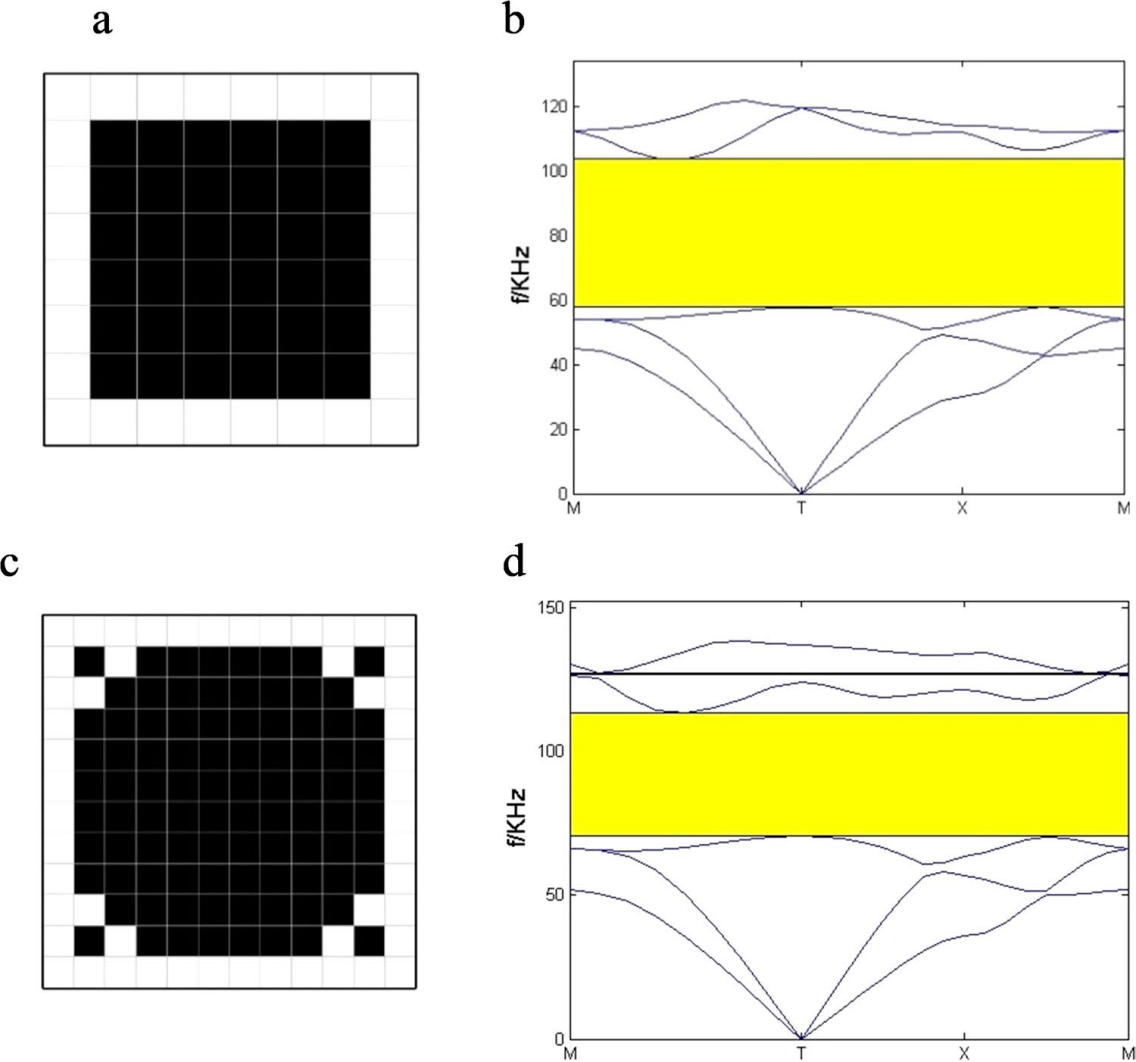
The topological optimization results for different N. N = 8: (**a**) the optimal configuration (**b**) the corresponding band structure. N = 12: (**c**) the optimal configuration (**d**) the corresponding band structure.

The optimization above is studied when N = 10. For different N, the optimization results are calculated. As we can see in Fig. 5(a), the optimal unit cell for N = 8 is also a simple lattice structure with square scatterer. The corresponding filling rate of the scatterer is 0.56. The optimal RBG is 0.55 in Fig. 5(b). When N = 12, the optimal unit cell changes and the corresponding filling rate is 0.64. The optimal RBG is 0.51. With the calculations of band gap based on the obtained configurations with different element numbers, the wider band gap can be obtained when the element number is 10 × 10. So, we selected 10 × 10 as fixed mesh for optimization in current work. Fixed mesh strategy is widely used in shape and topology optimization, as shown in^52,53^.

**Figure 6:**
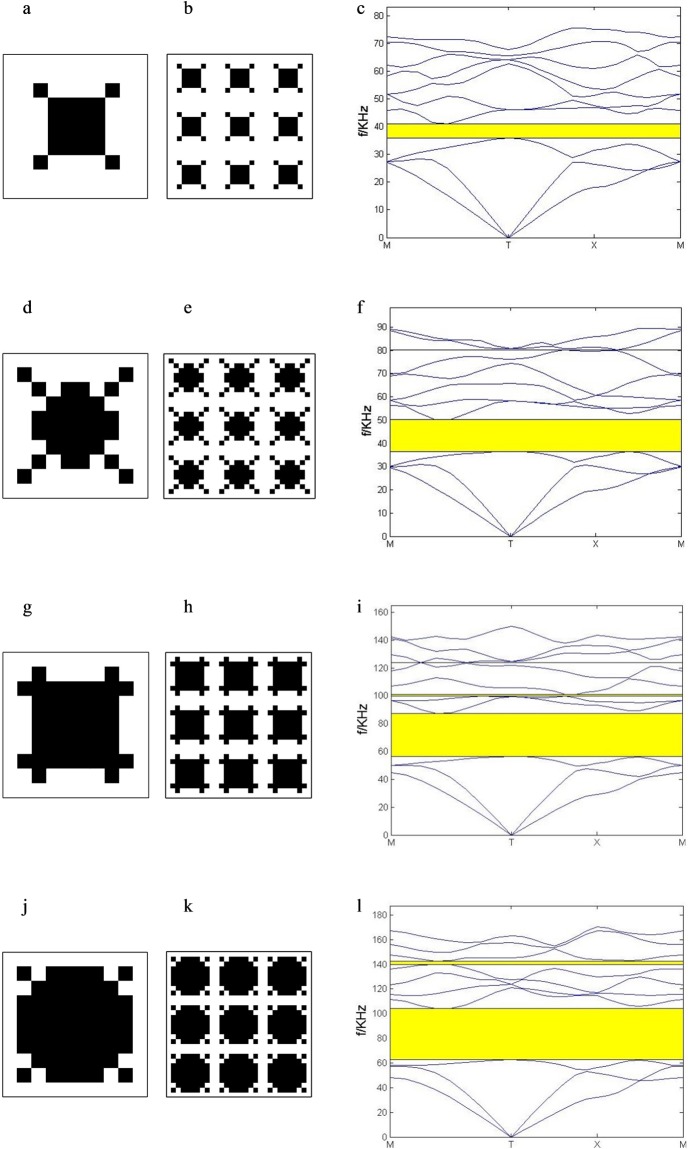
The opmization results for different filling rate. the topological configuration: (**a**) f = 0.2 (**d**) f = 0.32 (**g**) f = 0.44 (**j**) f = 0.56; the 3 × 3 unit cells: (**b**) f = 0.2 (**e**) f = 0.32 (**h**) f = 0.44 (**k**) f = 0.56; the corresponding band structure: (**c**) f = 0.2 (**f**) f = 0.32 (**i**) f = 0.44 (**l**)f = 0.56.

When the material of the substrate or the scatterer is limited, it is necessary to obtain wider BG by PC plate with adaptive configuration. The optimization including the optimal configurations, the corresponding 3 × 3 unit cells and the corresponding band structures of the thin PC plates for different filling rate are studied and given in Fig. 6. The filling rate f is selected at regular intervals. When f = 0.2, the optimal configuration of the scatterer is a square with corners along the diagonal in Fig. 6(a,b). The band gap is ranged from 35.9 kHz to 40.9 kHz and the RBG = 0.13 in Fig. 6(c). When f = 0.32, the band gap is ranged from 36.5 kHz to 50.2 kHz and the RBG = 0.32 in Fig. 6(f). When f = 0.44, the band gap is ranged from 56.3 kHz to 87.5 kHz and the RBG = 0.43 in Fig. 6(i). When f = 0.56, the band gap is from 62.5 kHz to 104.1 kHz and the RBG = 0.49 in Fig. 6(l).

**Figure 7:**
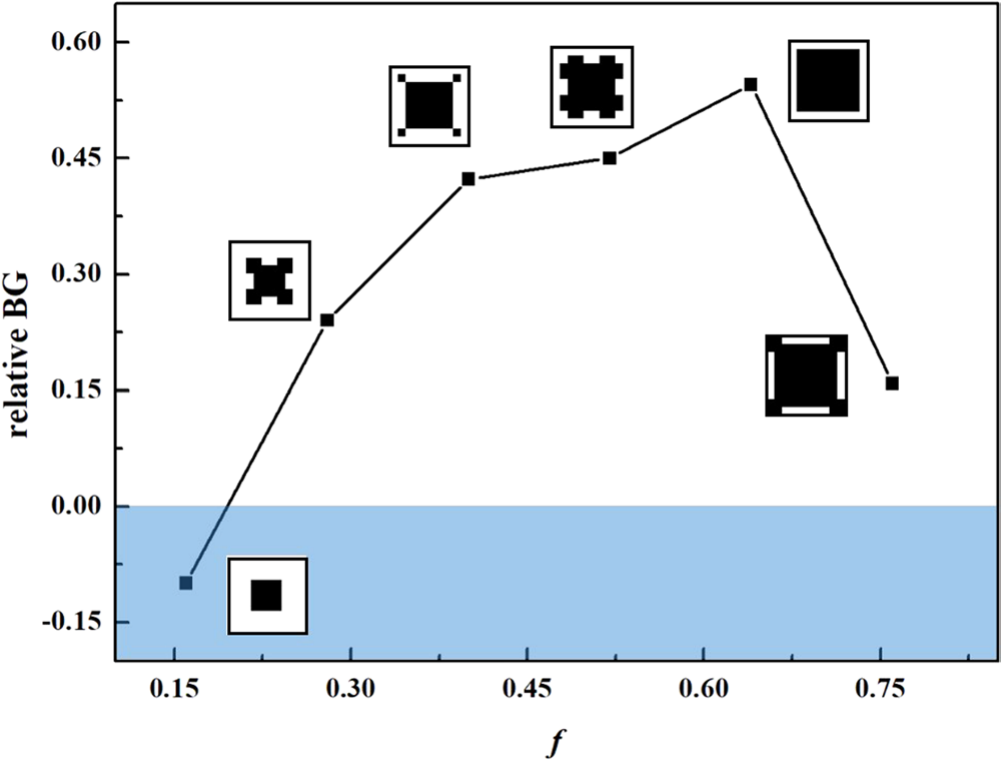
Variation of the RBG with the filling rate.

The variation of the widest RBG for different filling rate is discussed. As shown in Fig. 7, the RBG increases firstly when the filling rate increases to 0.64. In the case where filling rate is less than 0.64, the reflection and refraction becomes stronger because of the increasing contact area between the aluminium and the epoxy resign when the filling rate increase. However, when the filling rate increases, the interaction between the adjacent scatterers becomes stronger so that the pass band becomes wider and the band gap becomes narrower in the case where filling rate is greater than 0.64.

### The influences of the symmetry and material parameters

**Figure 8:**
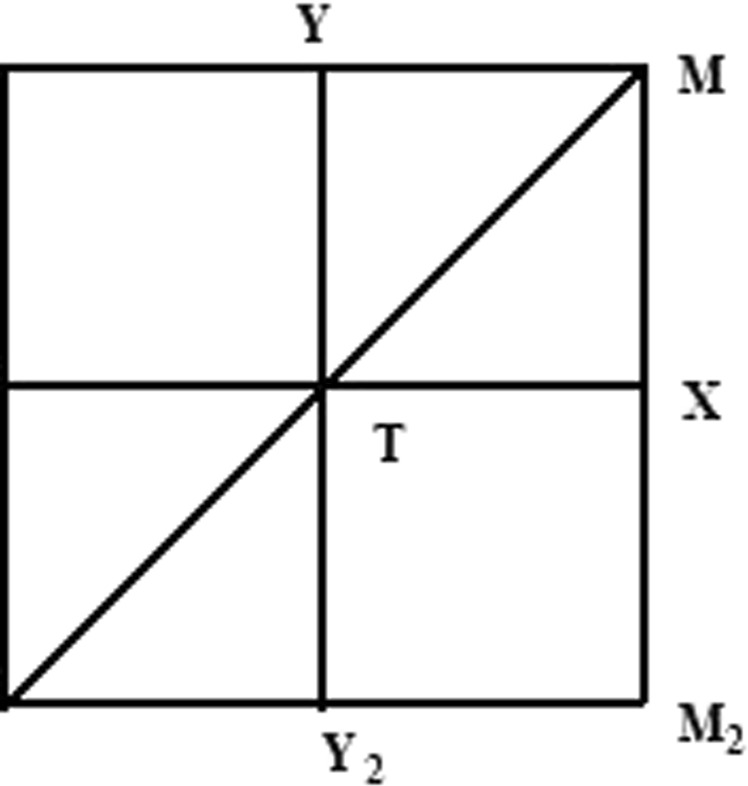
The first Brillouin zone.

**Figure 9:**
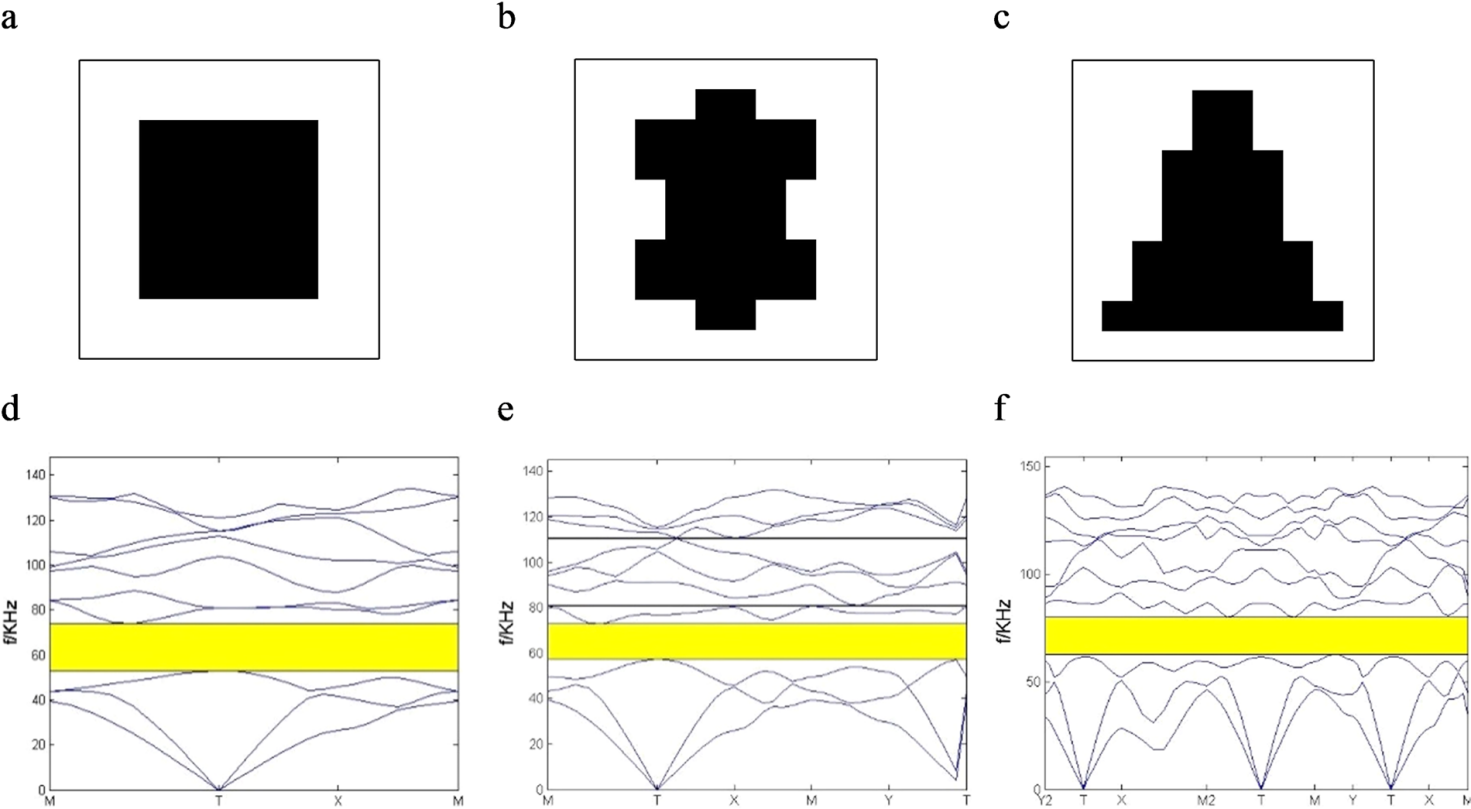
Variation of the RBG with the symmetry. The topological configuration: (**a**) four-axis symmetry (**b**) biaxial symmetry (**c**) uniaxial symmetry; The corresponding band structure: (**d**) four-axis symmetry (**e**) biaxial symmetry (**f**) uniaxial symmetry.

The variation of the RBG is discussed when the symmetry is changed. In initial optimization, filling rate is a parameter being selected to be state variable in topological optimization. Now, it is selected to be 0.36 randomly to study the effect of symmetry parameters on RBG. The first Brillouin zone is shown in Fig. 8. Three kinds of PCs with different symmetries are shown in Fig. 9 when filling rate is 0.36. For the four-axis symmetry PC in Fig. 9(a), the RBG is 0.33. The RBG of the biaxial symmetry PC in Fig. 9(b) is 0.27. For the uniaxial symmetry PC in Fig. 9(c), the RBG is 0.24. When the topological configuration of the filling material is highly symmetrical, wider band gap can be obtained. The width of the band gap decreases when it is symmetrical only along one or two axes.

**Figure 10:**
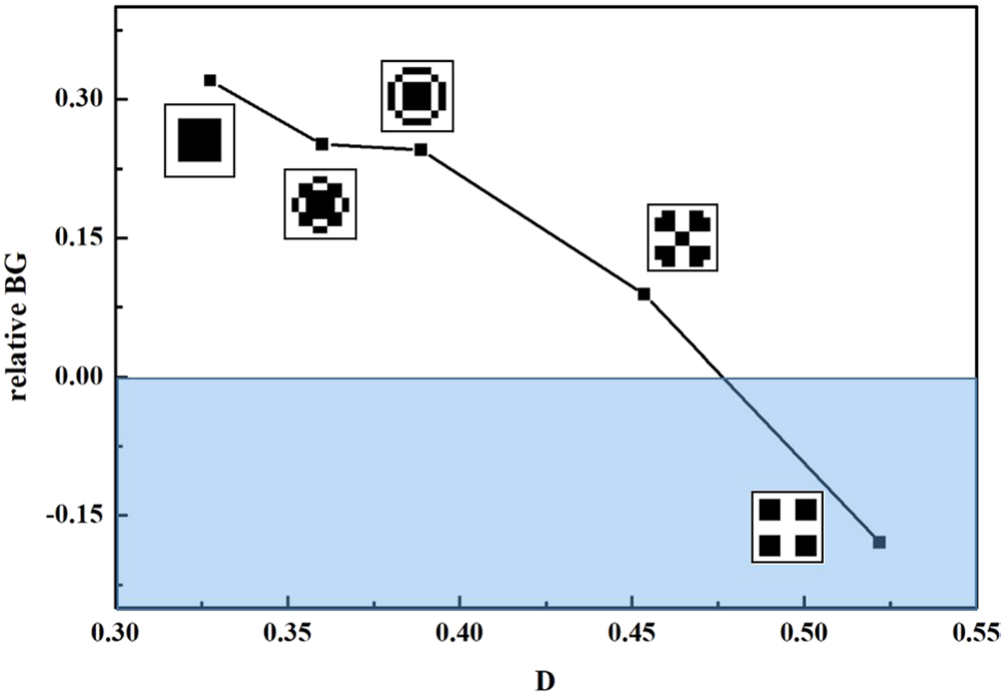
Variation of RBG with dispersion coefficient.

Define the coefficient of the dispersion as the quantity of the blank elements in the scatterer to represent the extent of polymerization of the PC. As shown in Fig. 10, when the coefficient increases, the relative band gap decrease. It has no band gap when coefficient of the dispersion increase to 40. The structure has good band gap only when the filling material is highly concentrated. When the coefficient of the dispersion increases, the distance between the adjust scatterers decreases. The interaction between the adjacent scatterers becomes stronger so that the pass band becomes wider and the band gap becomes narrower. At the same time, the scatterer effective density becomes smaller when the dispersion coefficient is increased in constant filling rate. BG width decreases when the density ratio between the scatterer and the substrate decreases.

**Figure 11:**
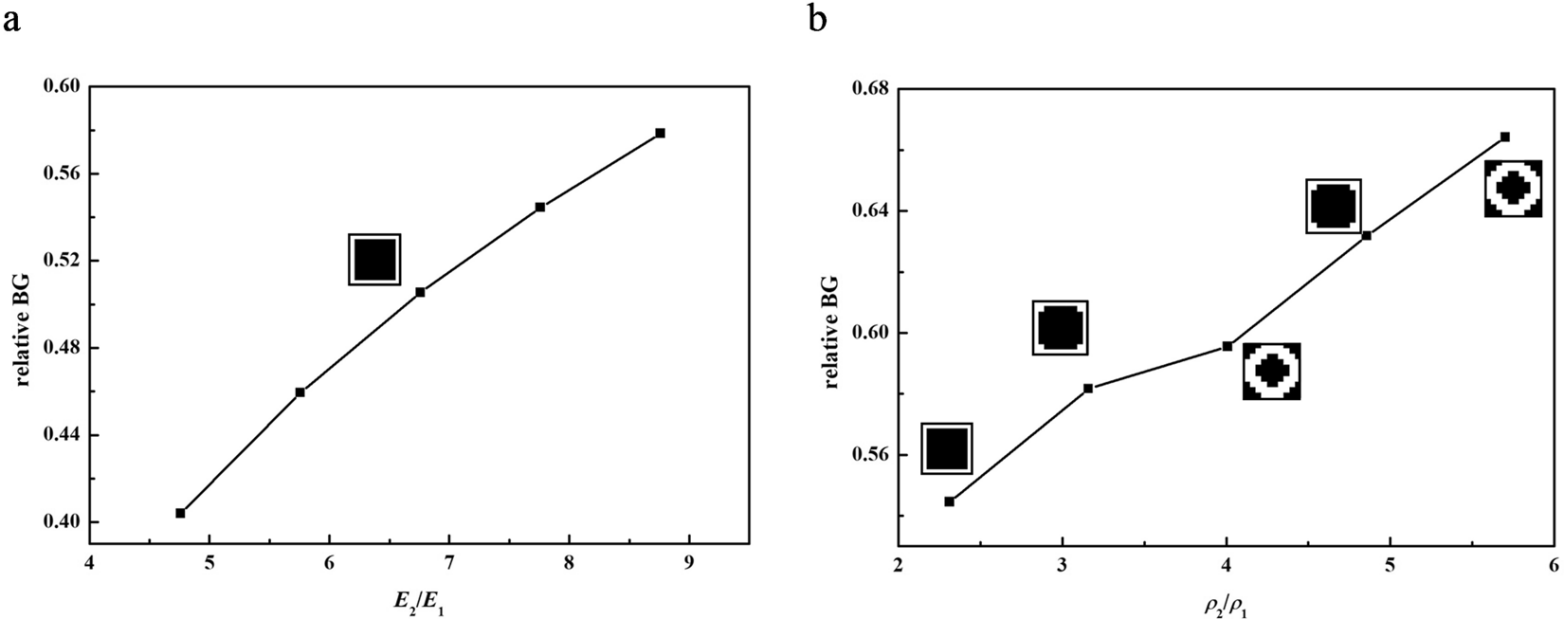
Effect of the material parameters on optimization results. (**a**) The relative Young’s modulus (**b**) the relative density.

The influences of the material parameters including Young’s modulus and density on the optimization results are considered when the filling rate is ignored. In Fig. 11(a), the optimal configuration which includes a square scatterer remains unchanged when the relative Yong’s modulus increases. The optimized relative band gap becomes wider because of the high contrast Young’s modulus. As shown in Fig. 11(b), the optimized relative BG increases when the relative density *ρ*_2_/*ρ*_1_ increases. The corresponding optimal configurations are different.

**Figure 12:**
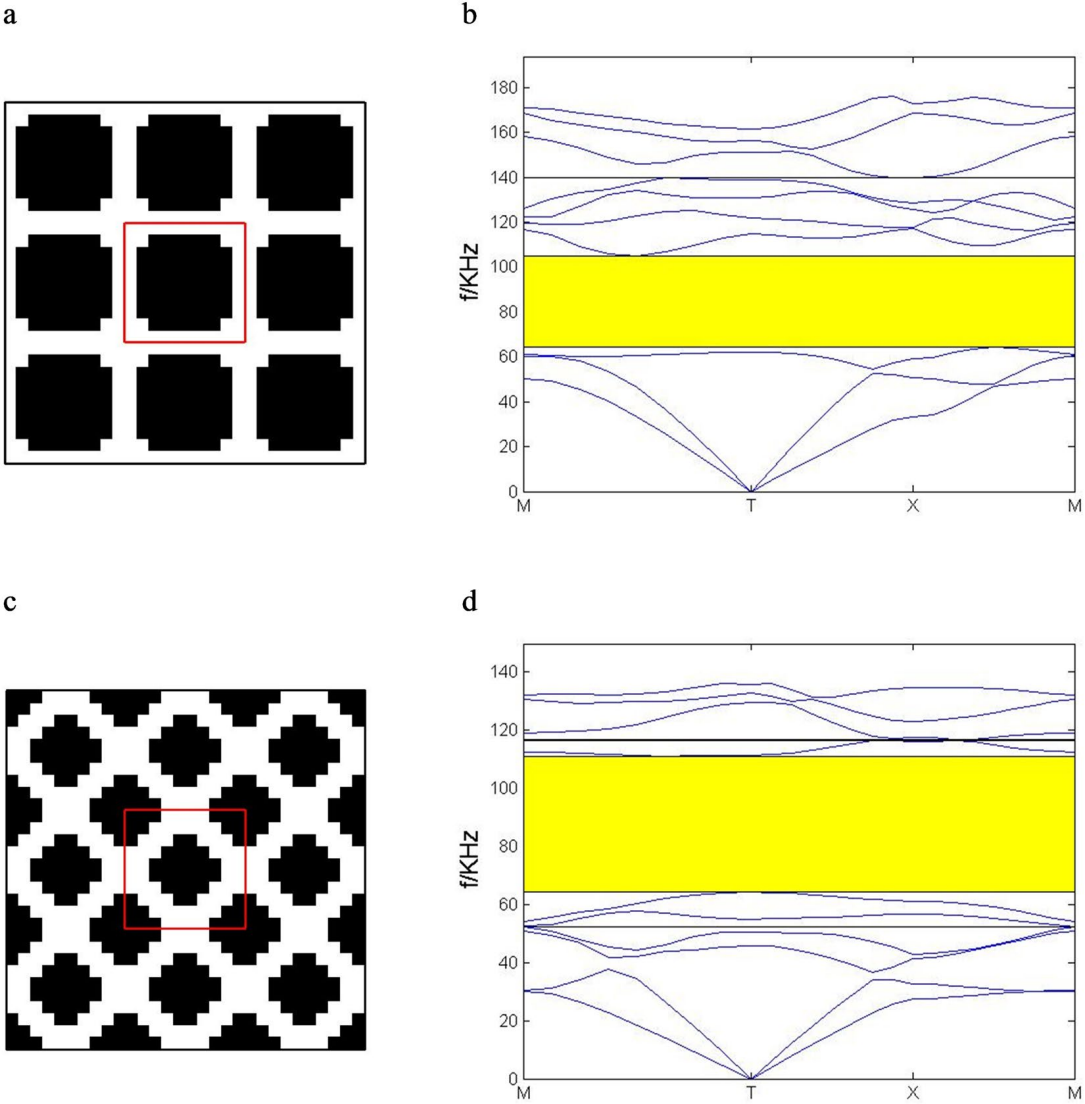
The optimization results for different relative density. *ρ*_2_/*ρ*_1_ = 3.2: (**a**) 3 × 3 optimal unit cells (**b**) the optimal band structure; *ρ*_2_/*ρ*_1_ = 4.0: (**c**) 3 × 3 optimal unit cells. (**d**) the optimal band structure.

When *ρ*_2_/*ρ*_1_ = 3.2, the scatterer is a square with missing corners as shown in Fig. 12(a). As we can see in Fig. 12(b), the band gap is located between the third and the fourth bands. As for the *ρ*_2_/*ρ*_1_ = 4.0, the optimal configuration consists a complete graph in center and quarters of the complete graph in four corners of the unit cell as shown in Fig. 12(c). The corresponding band gap is located between the seventh and the eighth bands in Fig. 12(d).

## Conclusions

In this paper, the PC thin plate with maximum RBG is designed by the genetic algorithm in combination with plane wave expansion method. Filling rates, symmetry, polymerization degree and material parameters are key factors for design of topological configurations and can lead to different topologies of phononic crystals. When the filling rate is ignored, the optimal configuration is a unit cell with a square scatterer that the filling rate is 0.64. For given filling rate, the optimization is also considered. The topological relative band gap increases first and then decreases when the filling rate increases. The optimal configurations are also different for different filling rate. The structure has good band gap only when the filling material is highly concentrated. The effects of the material parameters on the optimization are also considered. The discussions can provide an effective tool for the design of the PC thin plate with desired band gap.
